# Generative rules of *Drosophila* locomotor behavior as a candidate homology across phyla

**DOI:** 10.1038/srep27555

**Published:** 2016-06-08

**Authors:** Alex Gomez-Marin, Efrat Oron, Anna Gakamsky, Dan Valente, Yoav Benjamini, Ilan Golani

**Affiliations:** 1 Champalimaud Neuroscience Programme, Lisbon, Portugal; 2 Department of Zoology, Faculty of Life Sciences and Sagol School of Neuroscience, Tel Aviv University, Israel; 3 Cold Spring Harbor laboratory, NY, USA; 4 Department of Statistics and Sagol School of Neuroscience, Tel Aviv University.

## Abstract

The discovery of shared behavioral processes across phyla is a significant step in the establishment of a comparative study of behavior. We use immobility as an origin and reference for the measurement of fly locomotor behavior; speed, walking direction and trunk orientation as the degrees of freedom shaping this behavior; and cocaine as the parameter inducing progressive transitions in and out of immobility. We characterize and quantify the generative rules that shape *Drosophila* locomotor behavior, bringing about a gradual buildup of kinematic degrees of freedom during the transition from immobility to normal behavior, and the opposite narrowing down into immobility. Transitions into immobility unfold via sequential enhancement and then elimination of translation, curvature and finally rotation. Transitions out of immobility unfold by progressive addition of these degrees of freedom in the opposite order. The same generative rules have been found in vertebrate locomotor behavior in several contexts (pharmacological manipulations, ontogeny, social interactions) involving transitions in-and-out of immobility. Recent claims for deep homology between arthropod central complex and vertebrate basal ganglia provide an opportunity to examine whether the rules we report also share common descent. Our approach prompts the discovery of behavioral homologies, contributing to the elusive problem of behavioral evolution.

The establishment of homologies is an indispensable goal in evolutionary biology. In pre-Darwinian comparative anatomy, a homologue has been defined as “the same organ in different animals under every variety of form and function”^1^. Based on this definition, anatomists have compared skeletons using validated distinctions such as the forelimb, humerus, and radius, and compared brains using validated structures such as the thalamus, cortex, and striatum. These structures acquired their identity and validity as homologues by demonstrating the same relative position, connectivity, and morphogenetic history across a wide array of taxonomic groups^2^. The validity of these structures has been indispensable for establishing a rigorous science of anatomy. Similarly, the comparative study of behavior requires the identification of distinct elementary behavioral processes which, much like skeletal segments and neural circuits, could be established across a wide variety of taxonomic groups on the basis of their connectivity and moment-to-moment generative history^3^. The foundations for a comparative study can then be laid by selecting behavioral situations and measures that have a potential for generalizability across as wide a taxonomic group as possible. Such potential seemed to be available with cocaine induced locomotor behavior in *Drosophila melanogaster*.

The accumulation of detailed descriptions of arthropod^4^,^5^ and vertebrate movement^6^ makes the issue of shared principles of organization in the behavior of these taxonomic groups increasingly accessible for comparison. An opportunity for such comparison is offered by the report that, when treated with the dopamine reuptake inhibitor cocaine, *Drosophila melanogaster* performs a sequence of stereotyped behavior patterns, including locomotion and circling, that lead in and out of immobility, apparently similar to the sequence observed in cocaine induced locomotor behavior in rodents^7^,^8^. This remarkable similarity in the response to cocaine in flies and mice has led researchers to suggest that cocaine induced behavior was homologous in the two phyla. That same behavior has, at the same time, been portrayed as aberrant, unusual, and uncontrolled^9^, highlighting impairment in the functionality of the behavior. Note that while pointing out the striking similarity of the behavior induced by the drug in the two phyla was important, demonstrating a homology requires showing a relation of correspondence between parts of parts of larger wholes^2^,^10^. Therefore, in the general context of trying to find shared behavioral rules that would qualify as behavioral homologies, we revisited fly cocaine-induced behavior by collecting high-resolution kinematic data and examining them using the approach of comparative anatomy to the demonstration of homology. Our analysis leads us to suggest that the rules that shape fly cocaine-induced locomotor behavior are not only shared with rodent cocaine-induced locomotor behavior but also with intact locomotor behavior in vertebrates.

The idea that pathology, be it induced by drugs, lesions or genetics, can enhance and highlight fundamental structures otherwise hidden or incipient in the intact organism is as old as comparative anatomy. As early as 1830, to demonstrate the concept of unity of structure across the vertebrates, Goethe, being challenged by the absence of the pre-maxillary bone in man, demonstrated its presence in an infant with a hydrocephalus^11^,^12^. Recovery from brain damage and normal development of behavior have been compared to highlight features that would have stayed unnoticed in development: the voluntary use of the hand in people^13^,^14^,^15^,^16^, eating and drinking^17^, sexual behavior^18^, and locomotor behavior^19^. In flies, the active coordination between walking and trunk orientation has been highlighted by changing the coordination between these two directions with alcohol, and in a different way with cocaine^20^. Also here the drug-induced preparations enhanced and highlighted features of intact behavior that might have stayed unnoticed otherwise. We thus use cocaine administration as a convenient parameter or “knob” to “electrophorese” a behavioral process into its constituent parts, in order to test the behavioral homology hypothesis.

The seed for obtaining a phylogenetic perspective (or for missing it) is sown in the initial measurement phase, during the selection of measures made by the observer. This is perhaps why behavioral homologies have hardly been documented so far across distantly related species. Since selection of measures is inescapable, it might as well be made deliberately and explicitly, supported by a systematic justification, and on the basis of one’s particular aims. In our aim to establish *bona fide* behavioral homologies, we need to describe the set of generative rules that provide a formal definition for the coordinated interplay between the key kinematic measures that shape the animal’s locomotor behavior. We made a selection based on the dynamics of three main degrees of freedom: centroid speed, path curvature and body rotation (change of trunk orientation in the horizontal plane). Such a low-level description reflects our aim by holding for both arthropods and vertebrates; other aims may justify other selections. These three measures have been demonstrated to be actively managed by the fly^20^, therefore implying control of perceptual quantities. While any selection of variables might be informative, kinematic quantities that are actively managed by the animal have a potential for defining cross-phyletic generative rules.

In this study we analyze the structure of fly locomotor behavior in its own right, while at the same time using a methodology and measures enabling a cross-phyletic comparison. By concentrating on the morphogenesis of cocaine-induced behavior, we seek to reveal the generative rules^21^,^22^ that shape a substantial component of the locomotor behavior of both flies (see results) and vertebrates (see discussion and references therein). The generative rules provide a formal definition for the coordinated interplay between three key kinematic degrees of freedom that shape the animal’s locomotor trajectory. We test the hypothesis that the rules that shape cocaine-induced locomotor behavior are shared between flies and rodents. We first show that such generative rules in fruit flies consist of a narrowing down of the animal’s locomotor repertoire in a prescribed order during the transition into immobility, and a buildup of the repertoire in exactly the opposite order in the transition to full-blown intact behavior. We then discuss, based on previous work, that the same rules of narrowing down and buildup apply to dopamine-induced locomotor behavior in rodents and, most important, also represent a behavioral homology in intact locomotor behavior in vertebrates.

Recent claims for a deep homology between the arthropod central complex and the vertebrate basal ganglia^23^ provide an opportunity to examine whether the shared rules we propose can be supplemented with a historical perspective of common descent. Both narrowing down and buildup of the repertoire are action selection functions, both are exhibited upon cocaine administration (which is a dopamine re-uptake inhibitor), and both the central complex^24^,^25^,^26^,^27^,^28^,^29^,^30^ and basal ganglia^31^,^32^,^33^ mediate dopamine-induced behaviors. The shared generative rules can provide a specification of the demand^34^,^35^ on the neural activity and network connectivity within and between substructures of the central complex and the basal ganglia. For example, the buildup of a vertebrate’s locomotor repertoire, which has been recently attributed to dopaminergic feedforward loops operating in the basal ganglia^36^,^37^, can guide a study of the relations between the central complex and the buildup of locomotor behavior in arthropods during the transition out of immobility. One must, however, be cautious in drawing connections between putative homologies at different levels of organization from neural structures to behaviors. As Katz and coworkers have compellingly demonstrated^38^,^39^,^40^,^41^, although the coupling between most behaviors and the neural circuits that mediate them is highly conserved, natural selection can act separately and differently on these two levels of biological organization.

## Results

### Narrowing down of the path spatial spread into immobility and its buildup to normal behavior

**Figure 1:**
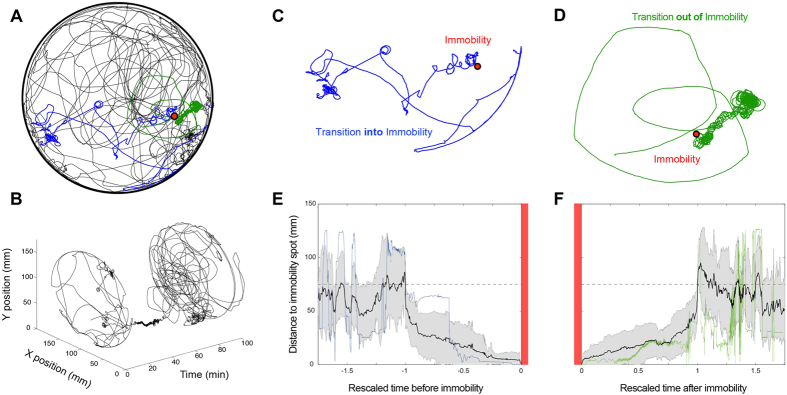
The locomotor path of cocaine treated flies progressively narrows down into immobility and builds up to spread out normal locomotor behavior. (**A**) A fly’s path for the entire 90 min session in a circular arena. Red dot indicates location of immobility. Blue path depicts transition into immobility, and green transition out of it. The rest of the trace throughout the experiment is represented in black. (**B**) Same path as in (**A**) unfolded in time. Transition into and out of immobility is clearly visible, and it is used as the point of reference to study the phenomenon. (**C**) The transition into immobility (corresponding to the blue path in A) is marked by the performance of straight paths, and then by increasingly more curved paths, narrowing down the spatial spread of the animal’s path. (**D**) The transition out of immobility (corresponding to the green path in A) is marked by the performance of curved, then increasingly straighter paths, building up the spatial spread of the animal’s path. (**E**) Distance of each fly to its corresponding immobility spot as a function of rescaled time. The average trend of activity (black line presents the mean for all flies, grey area depicts SEM) reveals a consistent narrowing down of the path during transition into immobility (alignment marked by the vertical red bars), and (**F**) a buildup of the path for all flies during transition out of immobility. In (**E**,**F**) blue and green traces correspond to the example from A-D. Despite the fact that single fly variability is high further away from immobility, the closer to immobility (both before and after) the more progressive and slow the spatial spread dynamics become.

Figure 1 presents the path traced by a single fly walking in the arena throughout a 90 min session. Upon cocaine administration, a complex dynamics of movement leads to immobility, followed by full recovery of movement (Fig. 1). The path leading to immobility is colored in blue, and the path leading out of it is colored in green. The path traced in space (Fig. 1A) unfolds in time (Fig. 1B) highlighting immobility as the origin to which movement converges and from which it unfolds. The fly first traces relatively straight paths, which become increasingly more curved culminating in immobility (Fig. 1C). Transition out of immobility starts with highly curved paths involving many tiny circles, followed by increasingly straighter paths (Fig. 1D). The progressive narrowing down of the path into immobility (Fig. 1E) and its progressive buildup back to normal (Fig. 1F) is quantified for all flies.

### Narrowing down of the fly’s locomotor repertoire and its buildup to a normal repertoire

**Figure 2:**
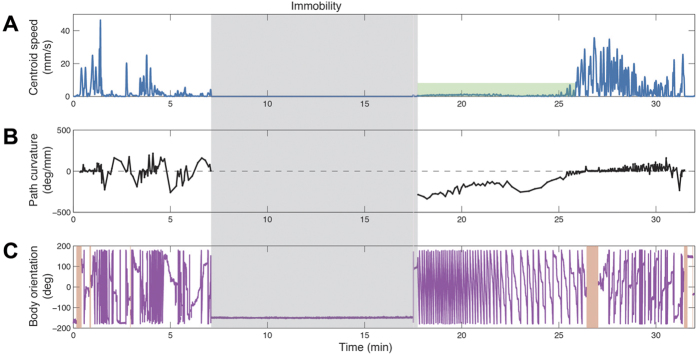
Representative moment-to-moment dynamics of the three kinematic degrees of freedom of a single fly before and after immobility. The shaded area marks the period of immobility, which is used as a reference for the events that precede and follow it. (**A**) The session starts with bursts of speed that progressively decrease towards zero. Following a 10 minute period of complete immobility, very low speed is then followed by normal speed. The green shaded area highlights small but non-zero velocity components at high-curvature during rotation in place (shown in detail in Fig. S2). (**B**) Straight path is followed by bursts of high curvature until immobility, from which the fly resumes its movement with very high curvature (of the order of a 360 degree turn in a millimeter) monotonically decreasing to straight paths again. (**C**) Extensive body rotations precede and follow immobility, proceeding from low to high frequency, and from high to low frequency. Red shaded areas represent time segments when the fly touches the walls of the arena and body orientation is not tracked. Each diagonal line represents a 360 degree body rotation. Overall, the session starts with extensive translation, then increasing curvature accompanied by frequent body rotations, leading into immobility. Following immobility, extensive rotation in place concurring with very high path curvature, is followed by forward progression along straight paths.

The three degrees of freedom of locomotor behavior studied are speed, direction of walking, and body orientation (Fig. S1 and Methods). Using extended immobility as a reference, we trace the behavior that preceded it (Fig. 2), starting just before the inflow of cocaine into the arena, and ending with full recovery of the fly (see Methods), as marked by the disappearance of high rotation in place (extensive changes in body orientation at low translational speed) and the performance of straight paths (high speed at low curvature). The entire dynamics in terms of speed (Fig. 2A), curvature (Fig. 2B), and rotation (Fig. 2C) are illustrated for a single fly. Transition into immobility started with normal locomotion marked by high speed, low curvature, and absence of extensive body rotations. Next, we observed bursts of high velocity followed by medium and then high path curvature, which concurred with the setting in of several full body rotations at very low translation speed, culminating in immobility. Immobile for 10 minutes (gray shaded area), recovery from immobility started with very fast whole-body rotations in place. Each diagonal line in magenta stands for a full 360 degree rotation. The fly performed approximately 50 full rotations in 10 minutes with almost zero translational velocity at extremely high path curvature. The high frequency of rotations gradually decreased, as did the path curvature (Fig. S2). Then the animal resumed normal forward progression involving relatively straight paths and high velocity bouts.

We can summarize the moment-to-moment dynamics observed as the following sequence: predominance of translation, then high curvature, and finally extensive exclusive rotation in place, ending in immobility, from which the same sequence is performed in reverse. Forward translation is thus eliminated from the fly’s repertoire first, and rotation last, in the transition into immobility (movie S1); while rotation is added to the repertoire first, and forward translation last, in the transition out of immobility (movie S2). Animations in time of the progressive narrowing down of degrees of freedom (movie S3) and of progressive buildup (movie S4) clearly represent the dynamics of the phenomenon under study.

### Coordination dynamics of fly walking direction and trunk orientation

**Figure 3:**
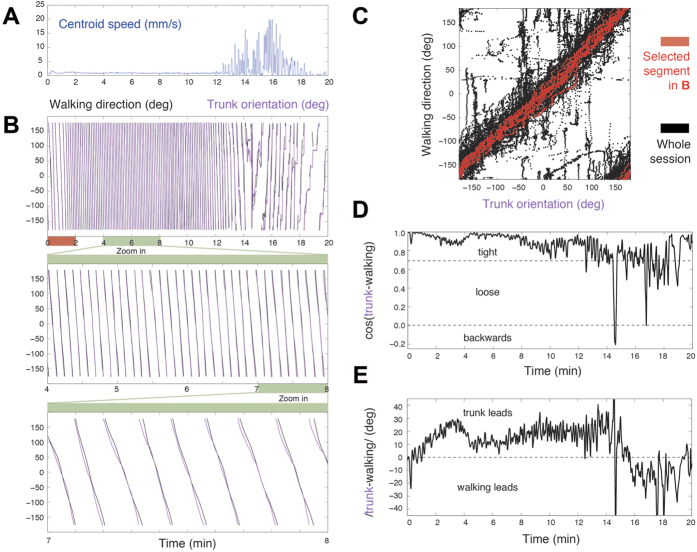
Active management of walking direction and trunk orientation. (**A**) Time course of centroid speed during transition out of immobility. (**B**) Walking direction and trunk orientation during intense rotation in place and high-curvature dynamics. A two-minute segment of the top plot is amplified in the middle plot; and a one-minute segment of the middle plot is amplified in the bottom plot, where the tight, but not perfect, coordination between both angles (trunk orientation and walking direction) can be appreciated. (**C**) Phase-plot of trunk orientation and walking direction value combinations across an entire session (black), and selected segment (red) showing that close to immobility the coordination is tight. (**D**) Cosine of the difference between trunk and walking angles, progressively decreasing from 1 (zero difference), to 0.7 (45 degree difference), and reaching below 0 (more than 90 degree difference), quantifying the transition from tighter to looser coupling of rotational and curvature degrees of freedom as the fly transitions out of immobility. (**E**) Signed angle difference between walking direction and trunk orientation to reveal the degree of freedom that leads and the one that follows: trunk orientation leads when close to immobility, with walking direction lagging behind a few degrees, and eventually leading at later stages in the buildup of locomotor behavior.

Flies can walk in different directions while keeping their body orientation fixed, or else reorient their trunk in any other direction while proceeding in a specific direction. In intact flies, during walking, the direction of progression changes first, and trunk orientation then converges to the new direction set by progression, with trunk orientation lagging behind by a small angular interval that is quickly closed. Under cocaine administration, trunk orientation lags on average behind by a larger angular interval, whose closing takes a longer time^20^. During the stage of transition out of immobility (Fig. 3A), as flies rotate along highly curved paths, the directions of progression and of trunk orientation tend to converge to the same value (Fig. 3B,C). The further away from immobility, the coupling between these two degrees of freedom becomes progressively less tight (Fig. 3C,D). As the fly regains its freedom of movement away from immobility, trunk orientation leads and direction of progression follows (Fig. 3E). In other words, the angular interval between the direction of progression and body orientation is modulated dynamically with respect to immobility and actively managed in two opposite ways.

### Generative rules of rotational-translational locomotor behavior in and out of immobility

**Figure 4:**
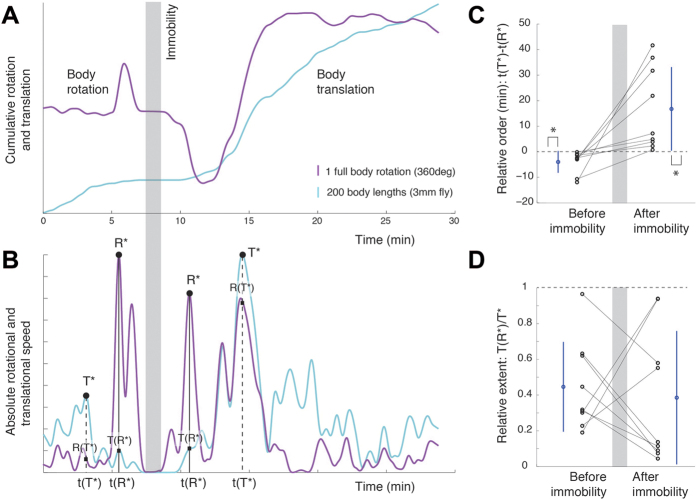
Synchronic and diachronic dynamics of translation and rotation. (**A**) Cumulative body rotation (magenta) and translation (cyan) reveal the global sequence of changes in the rotational and translational degrees of freedom. The shaded area marks the period of immobility, which is used as a reference for measuring the events that precede and follow it. (**B**) Global changes in speed of progression and body orientation are obtained from the absolute time derivative of the curves in (A). As shown, a global peak in translation followed by a global peak in rotation precede immobility, and a global peak in rotation followed by a global peak in translation follow immobility (TRIRT sequence). (**C**) Difference between the time of maximal peak of translation and the time of maximal peak of rotation, t(T*)-t(R*), is negative for transitions into immobility (p = 0.004, Sign test) and positive for transitions out of immobility (p = 0.004, Sign test). Absolute time differences are greater for transitions out of immobility than for transitions into immobility (p = 0.0352, Sign test). Each line connecting dots represents the same animal. Mean and standard deviation in blue. (**D**) Quantification of the strength of reciprocity in the value of global peaks of rotation and translation during transitions in and out of immobility shows hardly any difference. Dots represent the score for individual flies and lines connect the results for the same individual (mean and standard deviation colored in blue).

Having discussed the relationship between curvature and rotation, we concentrate on the relationship between translation (T) and rotation (R) with respect to immobility (I). Since the timescale we examine comprises the entire session dynamics (which can last for more than an hour), we next calculate cumulative translation by integrating speed in time and cumulative rotation by unwrapping the body angle (Fig. 4A). After smoothing the cumulative measures (see Methods) we calculate their derivative, and thereby obtain the global changes in rotation and translation for the entire session (Fig. 4B). In order to quantify the sequence in which they unfold, we again use immobility as a reference and measure the global peaks of activity before and after immobility, along each of the two degrees of freedom. This procedure reveals that before immobility the maximal peak of translation (T*) is exhibited before the maximal peak of rotation (R*). After immobility, it is the maximal peak of rotation that is exhibited before the maximal peak of translation. To quantify the relative order of global peaks, we calculate the difference between the time of maximal peak of translation and rotation, namely, t(T*)-t(R*). This procedure shows (Fig. 4C) that translation precedes rotation before immobility (average t(T*)-t(R*) is −4.0 minutes) and that rotation precedes translation after immobility (average t(T*)-t(R*) is +16.8 minutes). While there is high variability in the time intervals across animals, all flies follow the same sequential order of global peaks.

Next, in order to quantify the relative strength of the reciprocal relationship between global peaks, we calculate the value of translation at its peak, T(T*), and compare it with translation when rotation is at its peak T(R*). Thus we measure the amount of reduction in translation by the time that rotation is globally maximal. The smaller the ratio T(R*)/T(T*), the stronger the phenomenon of reciprocity between translation and rotation (Fig. 4D). Note that this relationship is by no means reciprocal at all times: in the presented graph there are cases in which both rotation and translation increase, and also in which both decrease together. In other words, rotation and translation are globally, not locally, reciprocal. Characterizing the relative strength of rotation and translation peaks by means of the above ratio is invariant to time rescaling and to absolute values of rotation and translation. This is necessary for capturing the invariance in the sequence and strength across individual animals, and particularly useful given the large variability in the timescales of unfolding of the phenomenon (some flies take minutes, others take hours) and in the rotation values (some flies perform ten full body rotations, others perform hundreds; and they do so at different rates).

On the whole, flies follow the same sequence of transition into immobility, involving, for each dimension separately, an enhancement, a reduction, and then elimination of that degree of freedom, thus progressively narrowing down the fly’s locomotor repertoire; and the same but opposite sequence of transition out of immobility, involving, for each dimension separately, an enhancement, and then subsiding to normal of that degree of freedom, thus progressively building up the fly’s locomotor repertoire. Such invariance can be summarized by the acronym TRIRT (generative rule consisting on the sequence: Translation, Rotation, Immobility, Rotation, and Translation).

### Rotational switching rate changes in and out of immobility

**Figure 5:**
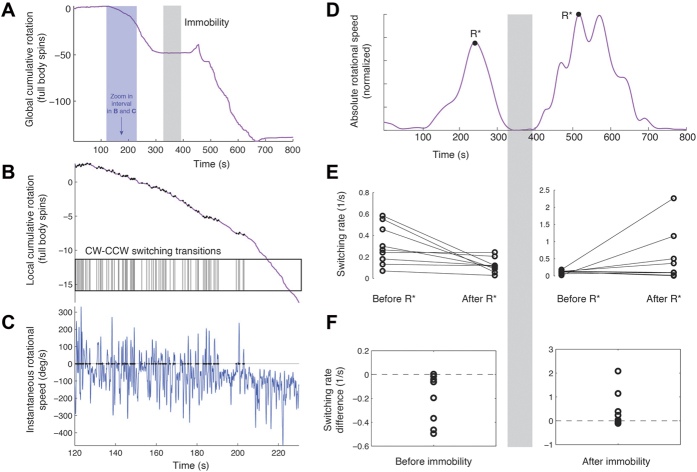
Switching between clockwise and counterclockwise rotation decreases into immobility and increases out of immobility. (**A**) Cumulative body orientation as a function of time for a single fly across the session. The gray shaded area marks the period of immobility. The blue shaded area marks a zoomed-in time interval presented in (**B,C**). As shown, the general tendency of this fly is to rotate clockwise both before and after immobility. Note that during transition into immobility the fly performs in the order of 50 full body rotations in 5 minutes, and during transition out of immobility, as many as 100 full body rotations in 5 minutes. (**B**) Zoomed in time segment in (A) of cumulative body rotation. As illustrated, while the fly globally rotates in one particular direction, locally it keeps switching between clockwise and counterclockwise rotations. Each transition is marked by a small black dot on the curve and a corresponding vertical bar on the transitions raster plot below. (**C**) Time derivative of the fly’s momentary body orientation. Zero crossings in the rotational speed mark switching, corresponding to the vertical bars in (**B**). As shown, there is an overall drift in one direction, concurrent with a decrease in the number of transitions as a function of time. (**D**) Global changes in body orientation obtained from the absolute time derivative of the curve in (**A**). To examine the change in switching across the session, the period leading to immobility is partitioned into the segments preceding and following maximal rotation (same for the period leading out of immobility). (**E**) Switching rate, calculated as the number of transitions per second, decreases in the interval preceding immobility (left panel, p < 0.039, Sign test) and tends to increase in the interval following immobility (right panel, not significant). Dots represent the score for individual flies and lines connect the results for the same individual. (**F**) Overall the difference of the switching differences after immobility and before it is always positive (p < 0.00195, sign test) and has a median of .37 switches per second. Dots represent the switching rate change for every fly.

One predominant effect of cocaine administration is the high rate of repetition of full body rotations and their rotational speed. During rotation the animal may switch between clockwise (CW) and counterclockwise (CCW) rotation directions. It is now possible to examine the dynamics of switching in reference to immobility, in the context of the animal’s freedom of movement. To do so, we focus on how frequently the fly changes the direction of rotation and how biased successive rotations are. Globally, there are long-term predominant biases to rotate in a particular direction (Fig. 5A). In particular, transitions out of immobility start by very long rotations in the same overall direction (we found no significant biases in handedness). However, flies do not rotate monotonically in one direction but, rather, alternate between large amplitude rotations in one preferred direction and low amplitude rotations in the other direction (Fig. 5B,C). Locally, the fly alternates between CW and CCW rotations. We find that the switching rate decreases when transitioning into immobility and increases when transitioning out of it (Fig. 5D–F). As found for the synchronic relationship between translation and rotation (TRIRT), this diachronic pattern for rotational switching also exhibits a mirror symmetry between the process leading into immobility and out of it (Fig. 5E–F), and can be summarized by the acronym SsIsS (sequence of high Switching, reduced switching, Immobility, and the reverse).

## Discussion

We characterized the generative rules that shape cocaine-induced behavior in Drosophila during transitions into and out of immobility. Using immobility as a reference for the measurement of behavior and cocaine as the parameter inducing a behavioral gradient, we found that flies exhibit a progressive buildup of their locomotor repertoire when starting from immobility, and a progressive narrowing down of the repertoire towards immobility. During buildup, for each key variable separately, the fly exhibits first enhancement and then reduction to normal values of movement along that variable: first, of body rotation in the horizontal plane, then of path curvature, and then of speed of translation. The extents of movement across the key variables show reciprocal relations: when rotation is at its peak translation is low, and when translation is at its peak rotation is low, while path curvature is partly coupled to rotation. Transition into immobility from rich normal locomotor behavior unfolds through narrowing down of the repertoire in the opposite sequential order, also showing reciprocal relations between the extents of the same variables. Quantification of the generative rules of this behavior, based on the temporal sequence of global peaks of extent (TRIRT, Fig. 4) provides a summary of the *bauplan* of this arthropod behavior, allowing a comparison with the rules reported in previous studies for movement into and out of immobility in vertebrates, in which the behavior has been termed “The Mobility Gradient”^34^.

Vertebrates and fruit flies share the same generative rules. *Buildup:* In infant mice transition out of novelty-induced immobility consists of side-to-side head movements that increase in amplitude, gradually recruiting the forequarters and then the hindquarters, to extensive rotation in place around the hindquarters. Only after exhausting the horizontal plane by rotating around the hindquarters, does forward stretching and subsequently forward translation appear, first along curved and then along straight paths (movie S5). The same sequence is exhibited in amphibians^42^, fish^43^, insectivores (movie S6), and carnivores^44^. Head-raising, forequarter-raising and, finally, rearing on the hind legs, are exhibited next^45^,^46^. The same sequence is exhibited both during moment-to-moment behavior and in ontogeny, during recovery from lateral hypothalamic damage^47^. Later on in development, during, for example, play and ritualized fighting interactions, the inferior animal exhibits the less mobile portion of the sequence, culminating in rearing and rotating around the hindquarters, whereas the superior may rear and rotate both around the hind-legs and around the forelegs, exhibiting an expanded freedom of movement in both the horizontal and vertical dimensions^44^,^48^ (movie S7); for a review see^19^,^34^. *Narrowing down*: The opposite sequence, proceeding from rich normal behavior to enhanced, then reduced, then immobility, first of rearing, then of translation along straight, and then along curved paths, then of rotation, culminating in relative immobility, is exhibited in rats following the administration of several dopamine agonists^7^,^8^,^49^,^50^,^51^,^52^,^53^ (movie S8). This mobility gradient^34^,^54^, which is composed of buildup (warmup) and narrowing down (shutdown) sequences^45^,^46^ shares with the mobility gradient demonstrated in fruit flies the same origin (immobility), parameter (dopaminergic stimulation; but in vertebrates also novelty and proximity to a rival), and generative rules.

As there are no “fossil skeletons” of behavior we suspend judgement regarding common descent. From the vantage point of comparative anatomy, generative rules define the core (or skeleton) that has resisted adaptation and around which there is a variable adaptive component. As shown by ethology, this core/adaptive distinction also applies to behavior^55^,^56^. The shared generative rules of the mobility gradient that we expose constitute a dynamical version of the “principle of connections” of St. Hillaire (“equivalence under transformation”^2^,^21^,^22^,^57^) whereby homology must be defined, not by its form or function, but by correspondence of relative positions, spatial structure, and, one might add, temporal relations between the elements of the behavioral process.

The “serial homology” concept can be applied when same structure serves different functions in the same species. Using our *generative rules* as a search image, not only can we predict essential sameness in the brain/behavior interface of both flies and rodents, but we can also anticipate that the same rules might underlie apparently different functional behaviors in the same species. This is similar to identifying serial homology in anatomy, where, e.g., hand and foot are considered homologous because they share the same set of developmental constraints, caused by locally acting self-regulatory mechanisms of differentiation^58^. Reviewing the fruit fly larval behavior literature with a search image for low and high mobility, attention is immediately drawn to the abnormally high extent of turning behavior exhibited by larvae with mutations in the gene *scribbler* (sbb) in the absence of food^59^. These appear to be respective manifestations of the high and low ends of the mobility gradient. The four key features characterizing low mobility in the cocaine-treated fly (low speed of translation, highly curved path, high body rotation, and immobility) exhibit a full correspondence to the features of the “abnormal crawling pattern” exhibited by *scribbler* larvae: low speed, curved paths, high turning rate, and long pauses^60^. The parameter precipitating this behavior could be, as Sokolowski and co-workers suggest, the absence of food, or else, given our search image, the stress brought about by the absence of food, or even its presence in hungry flies^61^,^62^. Equivalent differences in mobility, expressed by pivoting and/or rearing on hind legs and forelegs, reported to be exhibited by vertebrate partners engaged in interactions^44^,^48^,^54^, might also be looked for in fruit fly courtship and agonistic interactions.

Note that in this study we start with wild type behavior and end up with wild type behavior. By focusing on the behavior near immobility we thus highlight what intact flies do when away from immobility, namely *before* narrowing down, and *after* buildup, when they exhibit a full blown repertoire of locomotor behavior: alternating unpredictably between progression along straight paths, curved paths, wide and narrow circling, pivoting in place, and arrests, all within the constraints imposed by the specific environment which they occupy^20^. In addition, the mobility gradient model predicts that during agonistic and/or courtship interactions one fly would exercise freedom of movement in the vertical plane (rearing on its hind legs) and freedom to rotate (pivot) both around its hind legs and around its forelegs, while its partner would not rear, and pivot only around its hind legs.

When searching for equivalent neurochemical substrates that mediate the buildup and narrowing down, it has recently been claimed that the vertebrate basal ganglia and the arthropod central complex are deeply homologous. In both, comparable systems of dopaminergic neurons, their projections, and dopaminergic receptor activities are involved in the modulation and maintenance of behavior^23^. Dopamine systems are also key players in generating and regulating the mobility gradient^36^,^37^,^49^,^50^,^51^,^52^,^53^, and dopaminergic stimulation of specific substructures of the basal ganglia and central complex induce specific components of the mobility gradient respectively in rodents^63^,^64^,^65^,^66^,^67^ and in flies^68^. While studies of basal ganglia function typically focus on the adaptive component of behavior (habit formation), the narrowing down and buildup of the locomotor repertoire pertains to action selection within the hard core component, of which homologies are made: that which resisted change across evolution, all the more so that which does not change across ontogeny and across moment-to-moment behavior. The neurochemical processes mediating the buildup of the vertebrate locomotor repertoire have been recently attributed by Cools and co-workers to dopaminergic feedforward loops operating in the basal ganglia^36^,^37^. The correspondence between the mobility gradient core phenomena and the feedforward loops exposed in the basal ganglia can be used as a search image or working hypothesis for studying the relations between the arthropod mobility gradient and the central complex. It might be useful to examine whether feedforward loops also mediate the buildup of locomotor behavior functions in the central complex. It might be cautioned, however, that the validity of the shared generative rules does not depend on the establishment of deep homology at the neural level, since the nature of behavior does not necessarily dictate the neural mechanism mediating it and the presence of homologous neural components does not determine unequivocally the behavior^38^,^39^,^40^,^41^.

Many of the complexities of coordinated motor behavior in complex systems with many degrees of freedom can be derived from relatively simple biomechanical and non-linear mathematical laws^69^. Such laws, however, could not fully account for the bauplan that governs trans-phyletic locomotor behavior. The morphogenesis of behavior is not expected to be different from the morphogenesis of an anatomical limb bud, where the knowledge of how genes influence morphogenesis is not sufficient to construct a morphogenetic field model. For this we need to understand such principles as the physical laws describing viscoelastic media, stresses and strains, osmotic principles and how they act in the extracellular medium, and many other aspects of the macroscopic field of the limb bud that together provide a working model^22^. Be that as it may, the reservation suggesting concurrently distinct evolutionary processes at the neural and behavioral levels^41^, and the caution that simple physical laws are involved, along with genes and neurons, in shaping behavior^69^, only emphasize the requirement for a separate systematic study of connectedness^2^ at the level of behavior.

Given the foundational role of behavior in neuroscience^70^, it is perhaps time to start relating the various levels of the biological hierarchy to the behavioral *bauplan* they support. Here, practicing the methodology of comparative anatomy to the study of behavior, we have established a shared behavioral architecture across distant phyla based on the principle of connections. Demonstrating that such behavioral processes are respectively mediated by homologous neuro-genetic structures would endow the Mobility Gradient with the status of a Darwinian homology.

## Materials and Methods

### Fly stocks

Drosophila cultures were maintained at 24 °C on a standard cornmeal-molasses medium in 12 hour light/dark cycle at 60% humidity. The experiments were performed on three-day-old flies of the wild-type laboratory strain Canton-S. Nine male flies were tested in a low-throughput high-content data approach.

### Behavioral arena

The experimental setup for observing and tracking the flies was a 15 cm diameter circular arena with 0.7 cm height wall and transparent plastic ceiling. The arena was illuminated from a height of 70 cm above the arena level by flicker free incandescent light 40 W bulb (Osram). Throughout experiments we used a maximum-minimum thermometer (Brannan), which allowed us to ascertain that the temperature near the arena was maintained at 25 °C plus/minus 1 °C. A silent digital color camera (Sony Effio E Surveillance) placed at 70 cm above the arena recorded the fly’s behavior.

### Drug administration system

During the experiment there was a continuous airflow through the arena through two small wall openings allowing also the introduction of the volatilized cocaine from the evaporation chamber into the arena^20^. Continuous airflow, adjustable by a knob, supplied a mild stream of air into the arena at an intensity that maintained the cocaine white smoke inside the arena for one minute (increasing the airflow would immediately attract the fly to the pipe opening through which the air came in). The airflow was streamed into the arena continuously before and after the volatile cocaine administration, across the whole experiment. The drug volatilizing apparatus was connected to the arena by a short pipe. Cocaine was volatilized in a transparent, perspex chamber consisting of four volatilizing units. Each unit consisted of a nichrome wire connected to copper leads that were passed through a neoprene stopper and connected to a low voltage/high current regulated power supply^7^. Cocaine (150 ug) was volatilized from the nichrome filaments as follows. Free base cocaine dissolved in ethanol was applied to the filament and ethanol was allowed to evaporate. Evaporation of the cocaine was done using a low voltage/high current regulated power supply by applying a voltage sufficient to heat the filament to 200 °C within 5 seconds. A constant airflow through the arena removed the white cocaine smoke from the arena within one minute from start of administration. Cocaine was streamed into the arena, as ascertained by the white smoke filling the arena, until all the solid cocaine evaporated from the heated nichrome wires. Cocaine smoke does not condensate so that no residuals of cocaine were left in the arena following the experiment. The arena was washed with water and wiped with 70% alcohol after each experiment.

### Animal preparation

Neither food nor water was supplied to the fly during the entire experiment. All experiments were performed during the 12 hours light period, and on one fly at a time. Since different populations of flies differed in their reactivity to environmental stimuli but not in spontaneous activity^71^,^72^, we performed the experiment over an extended period of time so that drug treatment was given without disturbing the fly with the presence of other flies or with a novel environment, yielding spontaneous, rather than reactive, behavior^71^,^72^,^73^. Therefore, each fly was transferred to the arena and allowed to habituate there for one hour. Behavior was then videotaped for one more hour. Following exposure to cocaine, the fly behavior was videotaped for two more hours, in order to ascertain full recovery (regaining normal locomotor behavior).

### Behavioral tracking

The fly’s locomotor behavior was recorded at 25 frames per second at a resolution of 720 × 560 pixels (Sony Effio E Surveillance camera). Following video acquisition, the centroid position of the fly and its body axis direction were tracked with FTrack^74^, a custom-made software written in Matlab (MathWorks). The behavior of drug-treated flies was tracked and analyzed before the moment the drug started to be streamed into the arena chamber through the period of complete fly sedation (the longest arrest interval across the session), including the behavior following sedation until the fly proceeded along straight paths indicating full recovery of full blown intact locomotor behavior. Raw trajectory data were corrected for tilt and rotation of the camera. Data segments during which it was not possible to assess the fly’s orientation (fly located on the wall or jumping) were excluded from analysis.

### Behavioral analysis

Quantitative analysis of the animal’s behavior was based on the dynamics of three main degrees of freedom: centroid speed, path curvature and body rotation. Measuring the speed, the direction of progression and trunk orientation provided the three measured quantities^20^,^75^,^76^. In the Eshkol Wachman Movement Notation these variables amount to speed and direction of shift of weight, and direction of front^77^. Changes in the direction of progression are calculated per unit of progression and as a function of time in order to have a geometric curvature^78^,^79^ expressed in kinematic terms^80^,^81^. Switching between clockwise and counterclockwise body rotation was assessed via the zero-crossings of the instantaneous time derivative of the body angle, removing artifacts during arrests by pruning out rotations smaller than 12 degrees. Since we studied the same phenomenon across a wide range of timescales from small oscillations during rotation in place (seconds) to sedation recovery (minutes and hours) we obtained reliable estimates of local and global variables in time by using the variable-window smoothing LOWESS method^82^. Given the temporal resolution of the tracking, a simple sub-second filtering smoothed minute possible effects from pixel noise and putative illumination fluctuations. This allowed to detect immobility as absolutely no displacement at the same time that fast rotations in place and slower trends could be quantitatively captured with precision. Immobility was defined as the longest time interval of complete arrest across the whole session. Immobility duration, being variable across flies, was still much larger than the timescales typically observed for pauses between stop and go behavior in flies. The procedure of transforming chronological time into activity is key in revealing dynamical invariants despite animal-to-animal behavioral variability.

## Additional Information

**How to cite this article**: Gomez-Marin, A. *et al.* Generative rules of *Drosophila* locomotor behavior as a candidate homology across phyla. *Sci. Rep.* **6**, 27555; doi: 10.1038/srep27555 (2016).

## Supplementary Material

Supplementary Movie File 1

Supplementary Movie File 2

Supplementary Movie File 3

Supplementary Movie File 4

Supplementary Movie File 5

Supplementary Movie File 6

Supplementary Movie File 7

Supplementary Movie File 8

Supplementary Information
