# Supplementary material for: Generative rules of Drosophila locomotor behavior as a candidate homology across phyla
Source: Sci Rep. 2016 Jun 8;6:27555. doi: 10.1038/srep27555 (PMC4897781; doi:10.1038/srep27555)
Supplement: Supplementary Information [file srep27555-s9.pdf]

SUPPLEMENTARY INFORMATION

Generative rules of *Drosophila* locomotor behavior as a candidate homology across phyla

Alex Gomez-Marin, Efrat Oron, Anna Gakamsky, Dan Valente, Yoav Benjamini, and Ilan Golani

Supplementary Figures and Legends

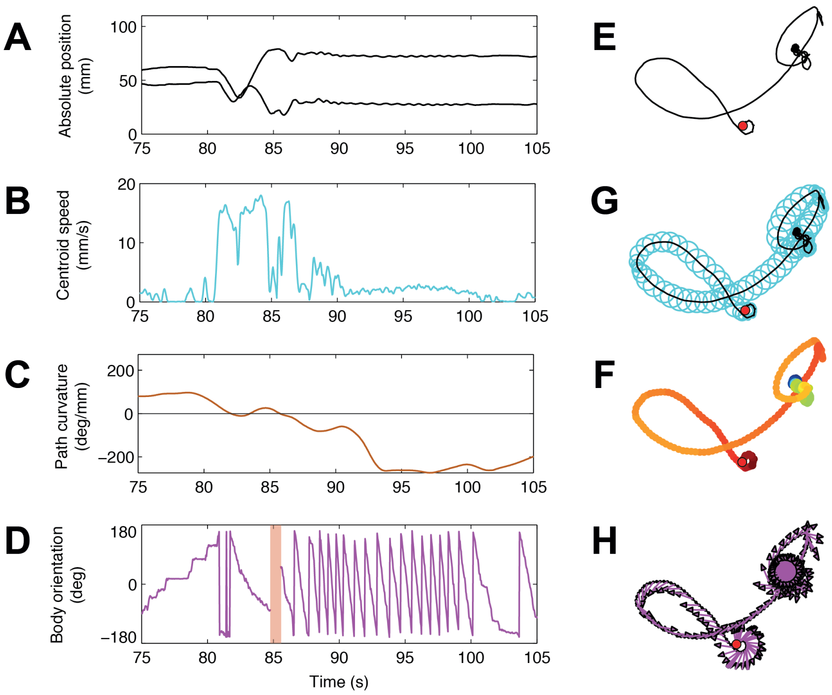

Figure S1. Three main kinematic degrees of freedom exercised by a fly at the trajectory level. Time evolution of x and y centroid positions (**A**), centroid speed (**B**), path curvature (**C**), and body orientation (**D**). Same degrees of freedom presented in space: black line represents fly path (**E**), disk diameter is proportional to speed (**G**), color coding depicts curvature (**F**), and arrows depict body orientation (**H**). Small red dot marks the beginning of the trajectory. During the 30 seconds time segment illustrated, a burst in speed as the animal traces a relatively straight path is followed by low speed at very high curvature, while the animal vigorously rotates in place, at approximately one full body rotation in place per second. Taking into account the orientation of the longitudinal axis of the fly's body (trunk orientation) as distinct from the direction of progression, we can access a third degree of freedom, thus distinguishing and quantifying how fast the fly is walking, in what direction it is moving, and in what orientation its body trunk is facing.

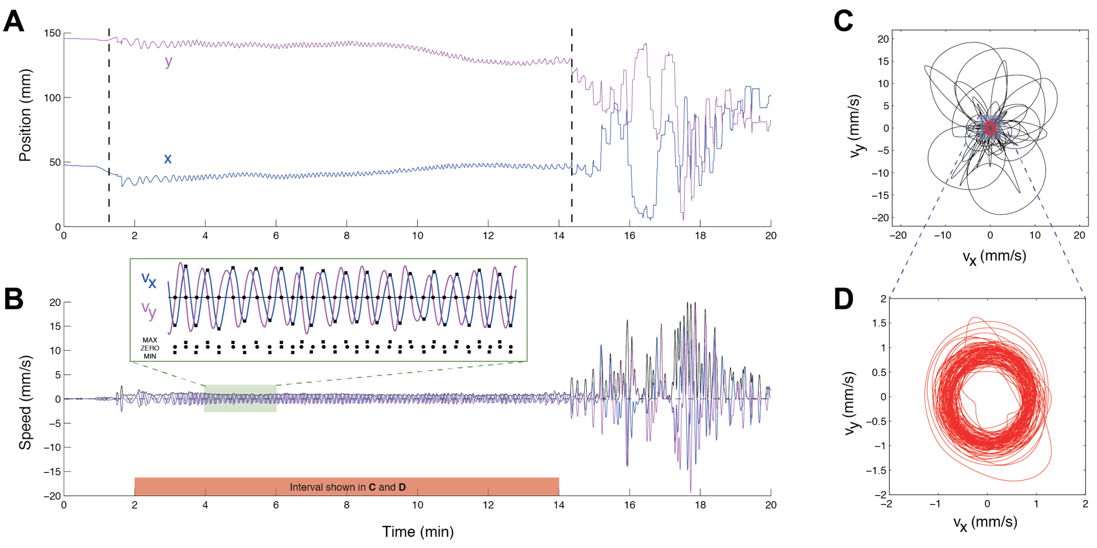

Figure S2. Detailed dynamics of the transition out of immobility based on velocity components, which determine speed and curvature and their coordination. (**A**) Transition out of immobility is illustrated by plotting the x and y positions as a function of time. Starting from absolute immobility, the fly performs tiny but fast oscillations in the x and y positions, reflecting fast rotations in place, which progressively slow down, and finally evolve into large displacements, corresponding to normal progression. (**B**) The early stage of transition out of immobility is characterized by very low speed, whose perpendicular velocity components (vx and vy) alternate in an oscillatory fashion (vx is zero when vy is max, and vice-versa; see inset in green), corresponding to very high curvature. The velocity components along the x and y directions capture the coordinated circling as the fly transitions out of immobility, whereas speed (v) misses the subtleties of rotation in place. (**C**) Phase-plot of speeds along x and y directions, containing both low speeds (in red), but also high-speed progression segments in all directions at a later stage of transition out of immobility. (**D**) Zoom in of the plot in (C) showing only the velocity components during the time interval from minute 2 to minute 14. On the whole, this closer look at path dynamics reveals that high curvature emerges from small, fast and alternating oscillations in orthogonal components of the velocity vector at typical speeds of 1mm/s, which reveal the minute circles traced by the animal as it rotates in place (during walking at higher speeds, flies show a much larger range of velocity components).

Supplementary Movies

Movie S1.
Link: https://www.youtube.com/watch?v=bgRyVv8Ae04
File: attached movie MS1_fly_NarrowingDown_track.avi
Legend: **Narrowing down of fly locomotion behavior upon cocaine administration (video tracking).** Upon sniffing cocaine Drosophila melanogaster performs a “shutdown” sequence, proceeding from forward progression to circling to rotating in place, culminating with immobility.

Movie S2
Link: https://www.youtube.com/watch?v=LBAugS1Rk7A
File: attached movie MS2_fly_BuildUp_track.avi
Legend: **Buildup of fly locomotor behavior upon cocaine administration (video tracking).** Upon recovery from cocaine influence Drosophila melanogaster performs a

“warmup” sequence, starting with rotation in place and then superimposing on it forward progression, thus generating circling. As rotation diminishes, circling turns into forward progression along relatively straight lines.

Movie S3
Link: https://youtu.be/CzT7sn0RdkM
File: attached movie MS3_fly_NarrowingDown_animate.mov
Legend: **Narrowing down of fly locomotor behavior upon cocaine administration (analysis animation).** Rotational versus translational degrees of freedom in Drosophila melanogaster narrowing down (“shutdown”) of locomotor repertoire within the Mobility Gradient upon cocaine stimulation.

Movie S4
Link: https://youtu.be/9vkTYuFQLSI
File: attached movie MS4_fly_BuildUp_animate.mov
Legend: **Buildup of fly locomotor behavior upon cocaine administration (analysis animation)**. Rotational versus translational degrees of freedom in Drosophila melanogaster buildup (“warmup”) of locomotor repertoire within the Mobility Gradient) upon cocaine stimulation.

Movie S5
Link: https://www.youtube.com/watch?v=wVaeqWPZnfc
File: attached movie MS5_infant_rat.mov
Legend: **Buildup of infant rat behavior upon novel environment.** When placed in a hostile environment outside its nest, an infant rat becomes immobile. Immobility is dissipated through the performance of horizontal movements that spread from head to tail. Forward progression is added after exhaustion of the horizontal plain, and finally vertical movement appears, here of the head. Vertical movement also spreads from head to tail.

Movie S6
Link: https://www.youtube.com/watch?v=3JQZgTwQrwM
File: attached movie MS6_hedgehog.avi
Legend: **Buildup of hedgehog behavior upon novel environment.** When placed in a hostile environment outside its home cage a hedgehog performs the warmup sequence: first pivoting in place, gaining support from forequarters to hindquarters and proceeding to forward locomotion.

Movie S7
Link: https://www.youtube.com/watch?v=YzD21jpa09g
File: attached movie MS7_rat_fight.avi
Legend: **Buildup and narrowing down of rat behavior upon social interactions.** A ritualized fighting interaction between two wild caught Rattus norvegicus males. The inferior animal exhibits the less mobile portion of the mobility gradient, culminating in rearing and rotating around the hindquarters, whereas the superior may rear and rotate both around the hind-legs and around the forelegs, exhibiting an expanded freedom of movement in both the horizontal and vertical dimensions.

Movie S8
Link: https://www.youtube.com/watch?v=ittpzspGZ_g
File: attached movie MS8_rat_drug.avi

Legend: **Buildup and narrowing down of rat behavior upon apomorphone administration.** Narrowing down of locomotor behavior under the influence of dopaminergic stimulation in a vertebrate. A bottom view of selected intervals from a one hour session of locomotor behavior of a laboratory rat injected with 1.25 mg/kg apomorphine chloride, a dopamine agonist. The video starts just before the drug starts to take effect, with the performance of a warmup sequence characterizing intact behavior: side-to-side forequarter movement and then pivoting around the hindquarters. Then, as the drug starts to take effect, the rat's locomotor behavior narrows down progressively: it first progresses along relatively straight paths, then along relatively curved paths which become progressively tighter, then pivots in place and then performs side-to-side horizontal (lateral) forequarter movements becoming increasingly immobile from hind- to forequarters.
